# Therapeutic inhibition of Mcl-1 blocks cell survival in estrogen receptor-positive breast cancers

**DOI:** 10.18632/oncotarget.27070

**Published:** 2019-10-09

**Authors:** Michelle M. Williams, David L. Elion, Bushra Rahman, Donna J. Hicks, Violeta Sanchez, Rebecca S. Cook

**Affiliations:** ^1^Program in Cancer Biology, Vanderbilt University, Nashville, TN 37232, USA; ^2^Department of Cell and Developmental Biology, Vanderbilt University, Nashville, TN 37232, USA; ^3^Department of Medicine, Vanderbilt University Medical Center, Nashville, TN 37232, USA; ^4^Department of Biomedical Engineering, Vanderbilt University, Nashville TN 37232, USA; ^5^The Vanderbilt-Ingram Cancer Center, Vanderbilt University Medical Center, Nashville, TN 37232, USA

**Keywords:** Mcl-1, ABT-263 resistance, mTORC1 signaling, luminal breast cancers

## Abstract

Cancers often overexpress anti-apoptotic Bcl-2 proteins for cell death evasion, a recognized hallmark of cancer progression. While estrogen receptor (ER)-α+ breast cancers express high levels of three anti-apoptotic Bcl-2 family members (Bcl-2, Bcl-xL, and Mcl-1), pharmacological inhibition of Bcl-2 and/or Bcl-xL fails to induce cell death in ERα+ breast cancer cell lines, due to rapid and robust Mcl-1 upregulation. The mechanisms of acute Mcl-1 upregulation in response to Bcl-2/Bcl-xL inhibition remain undefined in in ERα+ breast cancers. We report here that blockade of Bcl-2 or Bcl-xL, alone or together, rapidly induced mTOR signaling in ERα+ breast cancer cells, rapidly increasing cap-dependent Mcl-1 translation. Cells treated with a pharmacological inhibitor of cap-dependent translation, or with the mTORC1 inhibitor RAD001/everolimus, displayed reduced protein levels of Mcl-1 under basal conditions, and failed to upregulate Mcl-1 protein expression following treatment with ABT-263, a pharmacological inhibitor of Bcl-2 and Bcl-xL. Although treatment with ABT-263 alone did not sustain apoptosis in tumor cells in culture or *in vivo*, ABT-263 plus RAD001 increased apoptosis to a greater extent than either agent used alone. Similarly, combined use of the selective Mcl-1 inhibitor VU661013 with ABT-263 resulted in tumor cell apoptosis and diminished tumor growth *in vivo*. These findings suggest that rapid Mcl-1 translation drives ABT-263 resistance, but can be combated directly using emerging Mcl-1 inhibitors, or indirectly through existing and approved mTOR inhibitors.

## INTRODUCTION

The breast epithelium undergoes many dynamic changes throughout a woman’s lifetime. The intrinsic apoptotic pathway, which governs caspase-dependent cell death, is essential to maintain proper development and homeostasis during puberty, pregnancy, lactation, and post-lactational involution [1]. Not surprisingly, breast tumors frequently dysregulate this pathway to favor tumor cell survival, often through upregulation of anti-apoptotic Bcl-2 family proteins (Bcl2-A1, Bcl-2, Bcl-xL, Bcl-w, and Mcl-1) [2]. Anti-apoptotic Bcl-2 proteins bind to pro-apoptotic factors to prevent functional activation of the apoptotic pathway [3]. Specifically, anti-apoptotic Bcl-2 proteins either **1)** bind to Bcl-2 effectors (Bak and Bax) to block pore formation in the outer mitochondrial membrane caused by Bak/Bax oligomerization [4], or **2)** sequester Bcl-2 activators (*e.g.*, Bim, Bid, and Puma), which facilitate Bak/Bax oligomerization [5]. The dynamic and tightly regulated interactions between anti-apoptotic and pro-apoptotic Bcl-2 family proteins will tip the balance of cellular decisions towards or away from apoptosis.

Over 250,000 patients in the United States were diagnosed with invasive breast cancer in 2018, resulting in over 40,000 deaths. Estrogen Receptor (**ER**)-α positive breast cancer represents 60-70% of all breast cancers diagnosed. Notably, up to 70% of ER+ breast cancers express Bcl-2 [6], although Bcl-2 is expressed at low levels in other breast cancer subtypes [7, 8]. In contrast, Bcl-xL and Mcl-1 are widely expressed in ER+ breast cancers, as well as in *HER2*-amplified and triple negative breast cancers (**TNBC**) [7], both in pre-malignant lesions [9] and in high grade tumors [8]. Interestingly, Mcl-1 protein expression reportedly is higher in ERα-positive specimens [7, 10]. At the genetic level, *MCL1* is the most frequently amplified anti-apoptotic Bcl-2 family member in ER+ breast cancers [11]. Further, Mcl-1 protein expression correlates with poor patient survival in breast cancers regardless of subtype [12]. These observations support the intense research efforts into therapeutic targeting of anti-apoptotic Mcl-1 in breast cancers.

Because anti-apoptotic Bcl-2 family proteins neutralize pro-apoptotic effectors (Bak and Bax) and activators (Bim, Bid, and Puma) specifically through their Bcl-2 homology-3 (**BH3**)-domain binding pocket, a class of small molecular inhibitors that bind specifically within the BH3-domain binding pocket potently block interactions between anti-apoptotic proteins and their pro-apoptotic targets [13]. These ‘BH3-mimetics’ liberate BH3 motif-containing proteins (Bim, Bax, Bak, etc.) from interactions with anti-apoptotic Bcl-2 proteins, allowing pro-apoptotic effectors and activators to engage the intrinsic apoptotic pathway. BH3-mimetics targeting Bcl-2 and/or Bcl-xL have been successful as single agents in clinical studies of hematological malignancies [14–16]. However, single agent inhibition of Bcl-2 (using ABT-199) or dual inhibition of Bcl-2/Bcl-xL (using ABT-737 or ABT-263) was ineffective in pre-clinical models of human TNBC [10]. Similarly, studies in pre-clinical models of ERα+ breast cancers showed that ABT-263 was ineffective as a single agent, in large part due to rapid Mcl-1 upregulation [17], although the molecular mechanism(s) driving compensatory Mcl-1 upregulation in response to Bcl-2/Bcl-xL inhibition in ER+ breast cancers are not yet clearly defined.

Herein we show that increased Mcl-1 translation upon ABT-263 treatment drives survival of ERɑ+ breast cancer cells. ABT-263 treatment combined with a translation inhibitor, or combined with the mTOR inhibitor RAD001/everolimus, blocked Mcl-1 protein upregulation. Importantly, we found that the novel Mcl-1 small molecular weight inhibitor VU661013 blocked Mcl-1 activity in ER+ breast cancer cells, increased caspase-mediated apoptosis in ER+ tumor cells, and when used in combination with ABT-263, produced robust killing of ERα+ tumor cells in culture and *in vivo*.

## RESULTS

### Cap-dependent translation is required for Mcl-1 upregulation in response to Bcl-2/Bcl-xL inhibition

**Figure 1:**
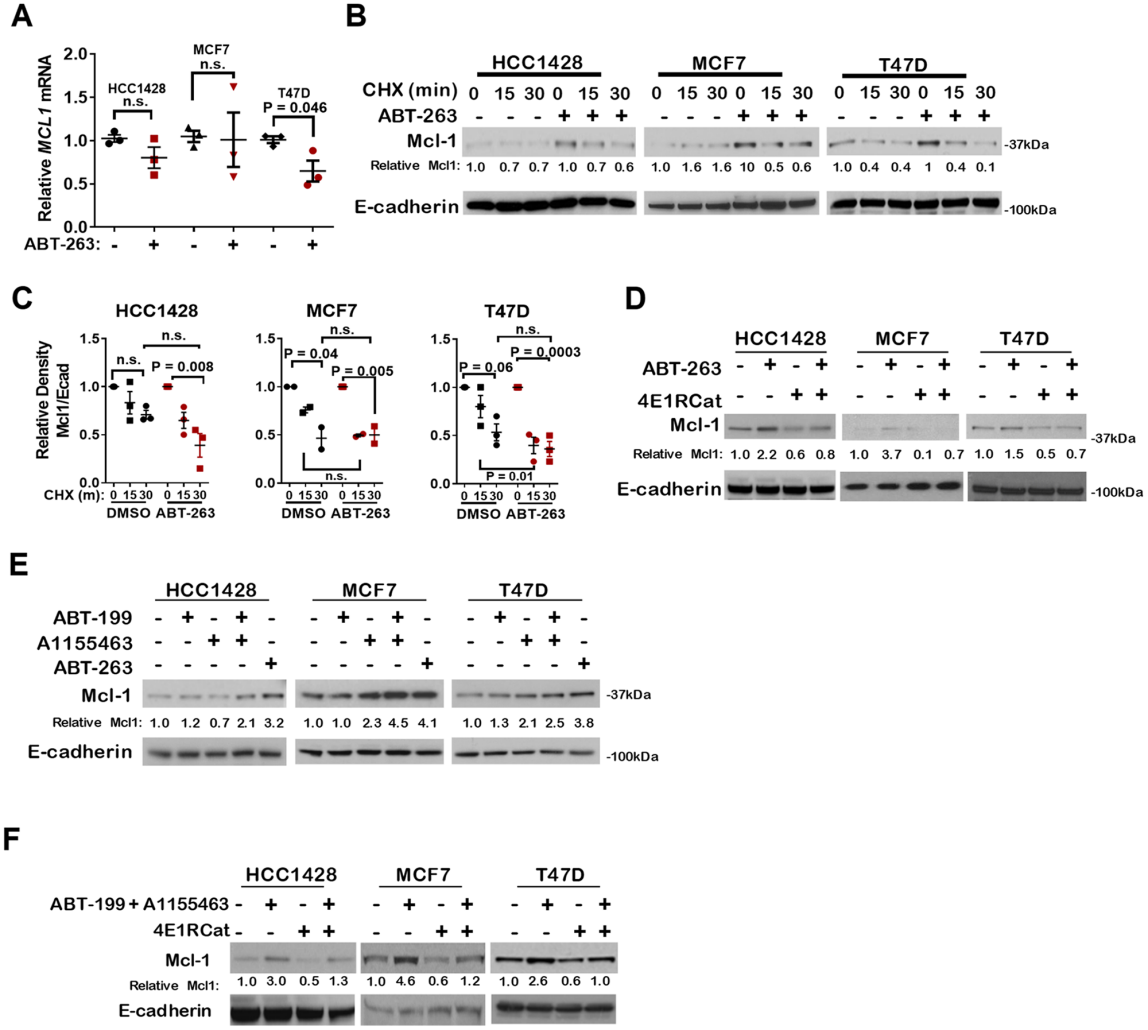
Pharmacological inhibition of Bcl-2 and/or Bcl-xL increases Mcl-1 expression through cap-dependent translation **(A)** Relative MCL1 transcript levels were determined by RT-qPCR after treatment with 1.0 µM ABT-263 for 16 hrs. Values were standardized to DMSO control for each cell line. Each data point represents the average of three technical replicates, midlines are the average of the biological replicates. P-value calculated using Student's unpaired two-tailed *t*-test. **(B–C)** Western analysis of lysates from cells treated with 1.0 µM ABT-263 for 16 hrs then chased with cyclohexamide (CHX) for 0-30 minutes. (B) Representative images are shown. Antibodies used are shown to the left of each panel. (C) Average Mcl-1 band density (± S.E.) is shown, N = 3. Two-way ANOVA and Bonferroni post hoc test. **(D–F)** Western analysis of whole cell lysates were harvested from cell treated with 1.0 µM ABT-263, ABT-199, A-1155463, or ABT-199 + A-1155463 for 16 hrs. Where indicated, 5.0 µM 4E1RCat was added for the final 4 hrs. Antibodies used are shown to the left of each panel.

Although combined inhibition of Bcl-2 and Bcl-xL using ABT-263 is capable of inducing apoptosis in ERɑ+ breast cancer cells [17], rapid Mcl-1 upregulation in response to ABT-263 limits the extent to which apoptosis is induced in these cells. To understand the mechanisms contributing to Mcl-1 upregulation upon combined Bcl-2/Bcl-xL inhibition, we treated ERɑ+ cells, including HCC1428, MCF7, and T47D, with ABT-263 (1 µM) or with vehicle control for 16 hours (**hr**), a time point at which Mcl-1 is upregulated in response to ABT-263. Relative *MCL1* transcript levels measured by quantitative real-time polymerase chain reaction (**qRT-PCR**) remained unchanged in HCC1428 and MCF7 cells treated with ABT-263, and were down-regulated in T47D cells treated with ABT-263 (Figure 1A), suggesting that transcript levels do not contribute significantly to changes in Mcl-1 protein levels upon ABT-263 treatment. Protein stability was assessed in cells treated with ABT-263 using cycloheximide (**CHX**) to block new protein synthesis. Mcl-1 protein levels assessed by western analysis revealed that Mcl-1 levels were upregulated in cells treated with ABT-263, as expected (Figure 1B). However, Mcl-1 diminution following CHX chase occurred at similar rates in cells treated with ABT-263 and in control treated cells (Figure 1B–1C). These findings suggest that Mcl-1 protein stabilization is not a major driver of Mcl-1 upregulation in response to ABT-263 in ERα+ breast cancer cells.

Increased cap-dependent translation of *MCL1*, particularly under conditions of cellular stress, reportedly contributes to Mcl-1 protein upregulation [18, 19]. This possibility was tested in ERα+ cells treated with ABT-263 using a pharmacological inhibitor of cap-dependent translation, 4E1RCat [20]. While Mcl-1 protein levels were increased in cells treated 16 hr with ABT-263, inhibition of cap-dependent translation with 4E1RCat blocked Mcl-1 upregulation following ABT-263 treatment in each cell line (Figure 1D).

Because ABT-263 blocks activity of Bcl-2 and Bcl-xL, both of which are expressed in ER+ breast cancer cells, we tested the impact of selective Bcl-2 inhibition versus selective Bcl-xL inhibition on Mcl-1 protein levels using the Bcl-2 selective inhibitor ABT-199 [16], and the Bcl-xL selective inhibitor A1155463 [21]. As expected, *MCL1* transcripts were not increased upon treatment with ABT-199 (1µM) or A1155463 (1µM) (Supplementary Figure 1A-1B), similar to what was seen in cells treated with the dual Bcl-2/Bcl-xL inhibitor ABT-263. Interestingly, western analysis did not reveal a pattern specifically implicating either Bcl-2 or Bcl-xL inhibition as a main driver of Mcl-1 upregulation across all three ER+ breast cancer cell lines tested (Figure 1E). Modest Mcl-1 upregulation was seen in HCC1428 and T47D cells treated upon Bcl-2 inhibition with ABT-199, but not in MCF7 cells treated with ABT-199 alone. MCF7 and T47D cells, but not HCC1428, increased Mcl-1 in response to inhibition of Bcl-xL using single agent A1155463. Importantly, combined inhibition of Bcl-2 and Bcl-xL using ABT-199 plus A1155463, or using the dual inhibitor ABT-263, induced Mcl-1 expression in HCC1428, MCF7 and T47D cells more robustly than inhibition of Bcl-2 inhibition alone or Bcl-xL inhibition alone. Mcl-1 upregulation seen in cells treated with the combination of ABT-199 and A1155463 was abrogated upon blockade of cap-dependent translation (Figure 1F), similar to what was found in cells treated with ABT-263. These findings are consistent with previous reports describing increased translation of Mcl-1 in Bcl-xL-impaired breast cancer cells harboring *PIK3CA* activating mutations (17), a mutation that increases signaling through phosphatidyl inositol-3 kinase (**PI3K**) and its downstream effector mTOR. However, we extend these findings to include cells that are *PIK3CA* wild-type (HCC1148), and in response to Bcl-2 inhibition. These results suggest that ERα+ breast cancer cells may rely heavily on cap-dependent translation of *MCL1* to drive rapid and potent Mcl-1 protein upregulation.

### Signaling through mTORC1 controls Mcl-1 protein upregulation upon loss of Bcl-2/Bcl-xL activity

**Figure 2:**
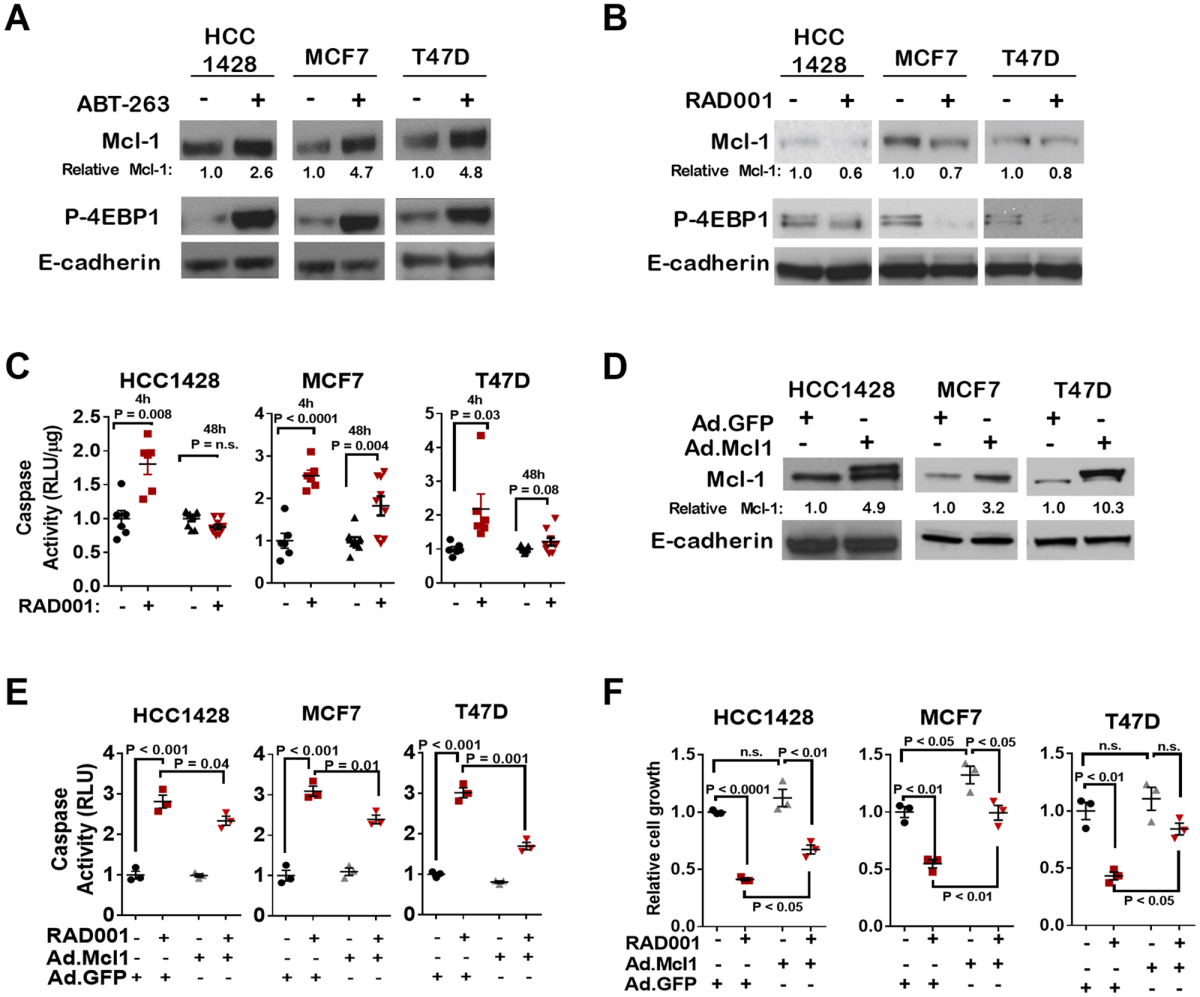
mTORC1 inhibition induces cell death through Mcl-1 depletion **(A)** Cells were treated with DMSO or with ABT-263 (1 µM) for 16 hrs, and whole cell lysates were assessed by western analysis using antibodies shown at left of each panel. Relative Mcl-1 calculated using densitometry analysis of Mcl-1 bands from western analysis films (corrected for E-cadherin densitometry analysis) using ImageJ software. **(B)** Cells were treated with DMSO or with RAD001 (200 nM) for 16 hrs (B) and whole cell lysates were assessed by western analysis using antibodies shown at left of each panel. Relative Mcl-1 calculated as described in panel A. **(C)** Caspase-3/7 activity was measured in cells treated ±RAD001 (200 nM) for 4h or 48h, and are shown as average (± S.E.) RLU/µg protein. Values are relative to the average value measured in DMSO-treated cells for each cell line. N = 6-9, Student's unpaired two-tailed *t*-test. **(D)** Western analysis of whole cell lysates harvested 48 hrs after transduction with adenoviral Mcl-1 (Ad.Mcl1) or Adenoviral GFP (Ad.GFP). Relative Mcl-1 levels were determined as described in panel A. **(E)** Caspase-3/7 activity was measured 72 hrs after adenoviral transduction. RAD001 (200 nM) or DMSO was added for the final 4 hrs, N = 3, each assessed in triplicate. Two-way ANOVA with Tukey's multiple comparisons test. **(F)** Single cell suspensions were seeded in 3D-matrix at 24 hr post-transduction, and cultured with DMSO or 200 nM RAD001 for 7 days. Average number of colonies/200x-field is shown for 3 experiments, each assessed in duplicate. All values for each cell line were set relative to the average number of colonies measured in Ad.GFP-transduced cells treated with DMSO. Two-way ANOVA with Tukey's multiple comparisons test.

Previous studies demonstrate that mTOR complex 1 (**mTORC1**) is a key regulator of protein translation, including cap-dependent translation, and as such is a dominant driver of tumorigenesis [as reviewed in [22]]. We tested the hypothesis that mTORC1 signaling may drive Mcl-1 upregulation upon Bcl-2/Bcl-xL blockade. We found that, in serum-starved cells treated with ABT-263 for 16 hr, phosphorylation of the mTORC1 substrate 4-Elongation Initiating Factor-Binding Protein-1 (**4EBP1**) was increased (Figure 2A), consistent with the notion that mTORC1 signaling may increase in response to Bcl-2/Bcl-xL inhibition. Notably, 4EBP1 is a key translational inhibitor whose activity is neutralized by mTORC1-mediated phosphorylation, thus enabling translation initiation to occur [23]. RAD001 (everolimus) [24], a rapalogue that inhibits the activity of mTORC1, was used to block mTORC1 signaling in ER+ breast cancer cells. In cells treated for 16 hr with RAD001 (200 nM), Mcl-1 protein levels were diminished as compared to what was seen in control-treated cells (Figure 2B). In addition to depleting Mcl-1 levels, RAD001 treatment for 4 hr increased caspase-3/7 activity in HCC1428, MCF7, and T47D cells (Figure 2C). Increased caspase 3/7 activity was maintained through at least 48 hr in MCF7 cells, but not in HCC1428 or T47D cells. Interestingly, adenoviral-mediated overexpression of Mcl-1 (Figure 2D) abrogated RAD001-induced caspase-3/7 activation over 4 hr of treatment (Figure 2E), and partially blocked RAD001-mediated growth inhibition over 7 days of growth (Figure 2F), suggesting that decreased expression of Mcl-1 is a major contributor to the growth inhibitory effects of mTOR inhibitors.

### mTORC1 inhibition depletes Mcl-1 expression and activity induced by ABT-263

**Figure 3:**
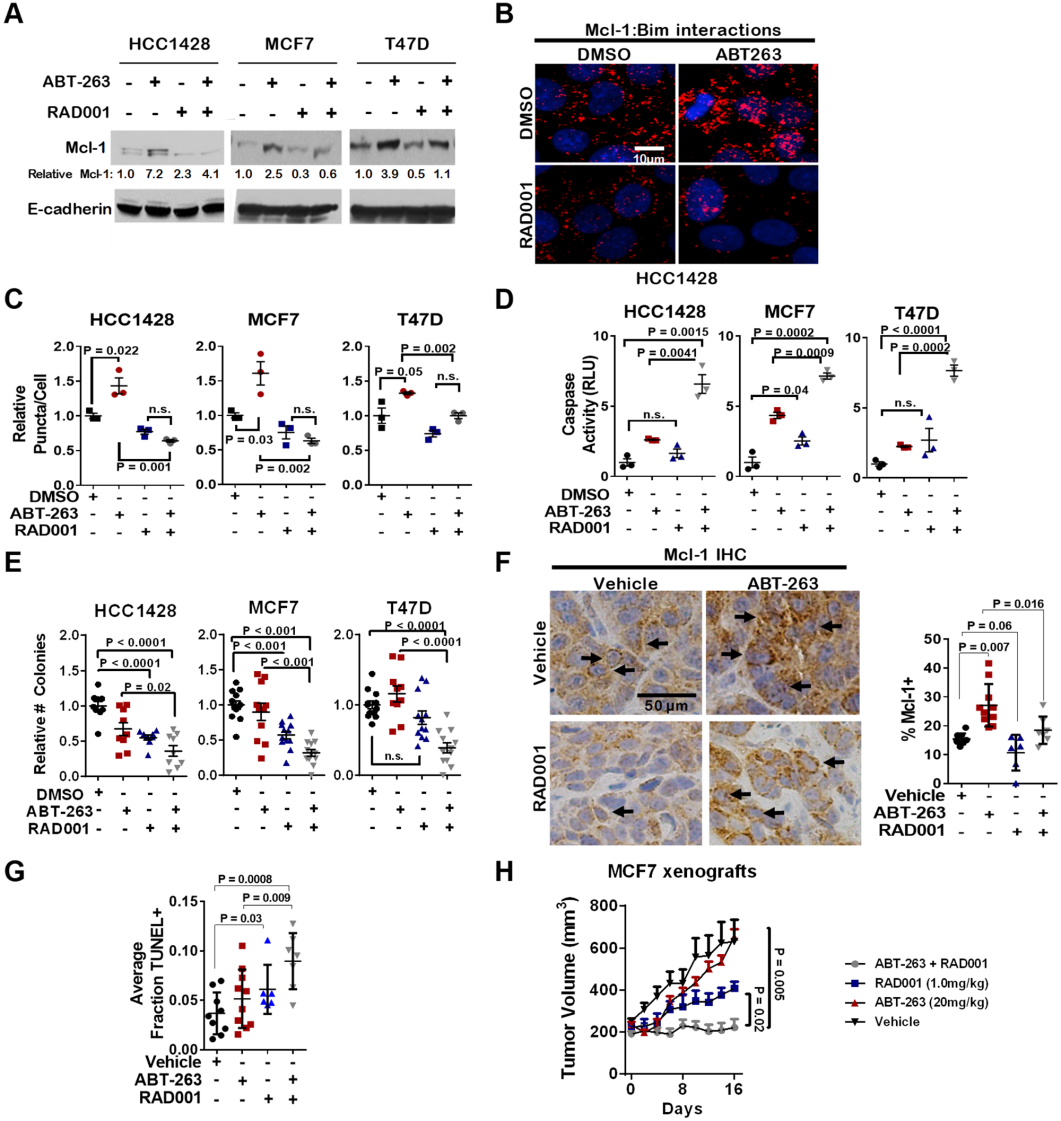
Inhibition of mTORC1 blocks Mcl-1 upregulation in response to ABT-263, and sensitizes ERα+ breast cancers to ABT-263-mediated cell death **(A)** Western analysis of whole cell lysates harvested from cells treated with 1.0 µM ABT-263 ± 200 nM RAD001 for 16 hr. Antibodies used are shown at left. Densitometry (Image J software) was used to determine relative Mcl-1 protein levels. **(B–C)** PLA using antibodies against Bim and Mcl-1 was conducted on methanol fixed cells after treatment with 1.0 µM ABT-263 ± 200nM RAD001 for 16 hrs. Representative images are shown (B) red puncta = Mcl-1:Bim proximity; blue = hoescht staining of nuclei. Quantitation is shown (C) Data points represent the average number of puncta/nucleated cell for 20 fields of view per sample, N = 3. Two-way ANOVA followed by Tukeys multiple comparisons test. **(D)** Caspase activity was measured in cells treated with 1.0 µM ABT-263 ± 200 nM RAD001 for 4 hr. Data points are the average RLUs corrected for total protein in three technical replicates, midlines are the average RLUs corrected for total protein of three biological replicates (±S.E.). The average RLUs in control cells for each cell line was set at a value of 1, two-way ANOVA followed by Tukeys multiple comparisons test. **(E)** Cells were cultured 7d in 1.0 µM ABT-263 ± 200 nM RAD001. Average relative cell number (±S.E.) is shown, N = 6-9, Two-way ANOVA followed Tukey's multiple comparisons test. **(F–H)** MCF7 tumor xenografts in athymic mice were treated with ABT-263 (20 mg/kg) and/or RAD001 (1 mg/kg) for 16 days. (F) IHC was used to visualize Mcl-1 expression on treatment day 16. Representative images are shown at the left, and the percentage of cells staining positive for Mcl-1 is shown at the right. Data points present the average in three fields per sample. Midlines represent the average across 6-9 samples per group. (G) TUNEL analysis was used to detect apoptotic cells, and quantitated as described in panel F. (H) Tumor volume of MCF7 xenografts were measured once every two days beginning on treatment day 0. Average tumor volume (S.E.) is shown. N = 6-9.

To explore the role of mTORC1 signaling in upregulation of Mcl-1 specifically upon treatment of cells with ABT-263, we treated cells with ABT-263 in the presence or absence of RAD001 for 16 hr. While ABT-263 induced Mcl-1 protein upregulation in ERα+ breast cancer cells, RAD001 abrogated Mcl-1 induction by ABT-263 (Figure 3A). A proximity ligation assay (**PLA**) detecting molecular proximity between Mcl-1 and Bim has been used previously as a measure of Mcl-1 activity [11, 25]. PLA confirmed increased Mcl-1 activity in ERα+ breast cancer cells treated with ABT-263, while showing that Mcl-1 activity was diminished in cells treated with RAD001, even in those cells treated with ABT-263 (Figure 3B–3C). Consistent with this observations, treatment of ER+ breast cancer cells with the combination of RAD001 plus ABT-263 increased caspase-3/7 activity (Figure 3D) and decreased cell growth (Figure 3E) to a greater extent than either inhibitor alone.

To confirm these findings *in vivo*, we treated mice harboring MCF7 xenografts with ABT-263 (20 mg/kg, daily) in combination with RAD001 (1 mg/kg, daily) for 16 days. Immunohistochemistry (**IHC**) demonstrated that phosphorylation of the mTORC1 substrate ribosomal protein p70 S6-kinase (**S6K**) was decreased after RAD001 treatment (Supplementary Figure 2), confirming inhibition of mTORC1 signaling. IHC detection of Mcl-1 protein expression in MCF7 tumors showed that while ABT-263-treated tumors upregulated Mcl-1 protein levels, RAD001-treated tumors displayed reduced Mcl-1 protein expression as compared to vehicle-treated or ABT-263 treated tumors (Figure 3F). Importantly, ABT-263-mediated upregulation of Mcl-1 was blocked by RAD001, which restrained Mcl-1 to baseline levels, further supporting the idea that ABT-263 treatment relies on mTORC1-mediated Mcl-1 upregulation, which can be blunted by mTORC1 inhibitors.

Terminal dUTP Nick-End Labeling (TUNEL) analysis of MCF7 xenografts collected at treatment day 16 showed that the combination of ABT-263 + RAD001 induced the greatest rate of tumor cell death (Figure 3G and Supplementary Figure 3), while tumor cell proliferation, measured by phospho-histone H3 staining, was unaffected by treatment with RAD001 or ABT-263 as compared to RAD001 alone (Supplementary Figure 4). Although ABT-263 alone (N = 10) did not significantly alter the growth of MCF7 tumors as compared to those treated with vehicle control (Figure 3H), RAD001 (N = 7) decreased MCF7 tumor growth by nearly 35% as compared to controls (N = 9). However, tumor growth was blocked using the combination of RAD001 and ABT-263 throughout the 16-day treatment period (N = 7), confirming that mTORC1 inhibition restrained Mcl-1 upregulation following Bcl-2/Bcl-xL inhibition, increasing the therapeutic effect of ABT-263 in ERɑ+ breast cancer xenografts *in vivo*.

### The Mcl-1 selective inhibitor VU661013 blocks Mcl-1 activity and induces apoptosis in ER+ breast cancer cells

**Figure 4:**
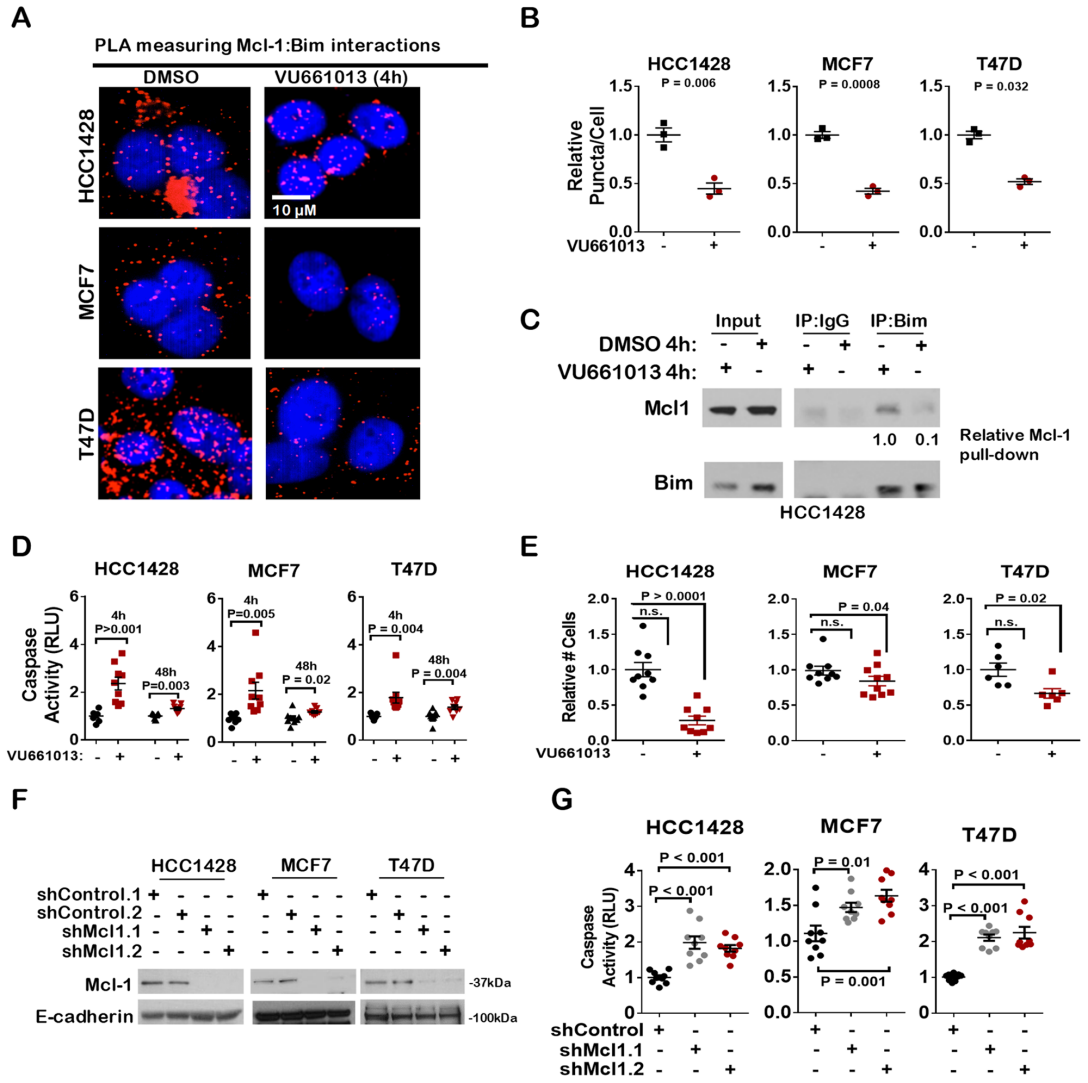
The novel Mcl-1 inhibitor VU-661013 bocks Mcl-1 BIM interaction and induces apoptosis in ER+ breast cancer cells **(A–B)** Proximity ligation assay (PLA) using antibodies against Bim and Mcl-1 was conducted on methanol fixed cells after treatment with 1.0 µM VU661013 for 4 hrs. (A) Representative images are shown. Mcl-1:Bim proximity, blue = hoescht staining of nuclei. (B) Quantitation is shown. Data points represent the average number of puncta/nucleated cell for 20 fields of view per sample, N = 3. Student's *T*-test. **(C)** Western analysis of whole cell lysates (input) or Bim immunoprecipates (IP: Bim) from whole cell lysates harvested from cells treated with 1.0 µM VU661013 for 4 hrs. Antibodies used for western analysis are shown at left. Densitometry (Image J software) was used to determine relative Mcl-1 protein levels. **(D)** Caspase 3/7 activity was measured in cells treated with 1.0 µM VU661013 for 4 hr and 48 hr. Data points are the average RLUs corrected for total protein in three technical replicates, midlines are the average RLUs corrected for total protein of 6-9 biological replicates (±S.E.). The average RLUs in control cells for each cell line was set at a value of 1, Student's *T*-test. **(E)** Cells grown were treated with 1.0 µM VU661013 for 7 d. Average relative number of cells per well (plus minus S.E.) is shown, N = 6-10, Student's *T*-test. **(F–G)** Cells were transduced with lentivirus expressing two distinct MCL1 shRNA sequences, or two distinct non-coding shRNA sequences, and selected with puromycin. (F) Western analysis of pooled clones was used to measure Mcl-1 protein expression. (G) Caspase 3/7 activity was measured in pooled clones of shRNA-expressing cells. Individual data points show the average of two technical replicates, N = 8-9 biological replicates.

The Mcl-1 selective inhibitor VU661013 previously was shown to induce tumor cell killing in pre-clinical models of TNBC and acute myelogenous leukemia [26, 27], but has not yet been tested in models of ER+ breast cancer. PLA confirmed that after 4 h treatment with VU661013 (1 µM), Mcl-1 interactions with Bim was substantially reduced to less than 50% of what was seen in control-treated cells (Figure 4A–4B). These findings were confirmed by assessing co-precipitation of Bim with Mcl-1. While Bim co-precipitation with Mcl-1 was seen in control-treated cells, the interaction was blocked in HCC1428 cells treated 4 hr with VU661013 (Figure 4C and Supplementary Figure 5). Consistent with blockade of Mcl-1 activity, treatment of cells for 4 hrs with VU661013 increased Caspase 3/7 activity in ER+ breast cancer cells (Figure 4D). Caspase activity remained modestly, but significantly, elevated through 48 hr of treatment with VU661013. Additionally, growth of cells in monolayer over 7 days was diminished upon treatment with VU661013 (Figure 4E). These results are similar to what was seen in ER+ breast cancer cells expressing short hairpin RNA (shRNA) sequences against *MCL1* (shMcl1) (Figure 4F), which displayed increased Caspase3/7 activity as compared to cells expressing non-targeting control sequences (shControl) (Figure 4G). These results are in agreement with previous reports demonstrating that Mcl-1 knockdown increases caspase-dependent apoptosis in ER+ breast cancer cells, and confirms that the Mcl-1 inhibitor VU661013 reproduces the phenotypic effects of gene-specific Mcl-1 knockdown.

### VU661013 combined with ABT-263 increases tumor cell death to a greater extent than either agent alone

**Figure 5:**
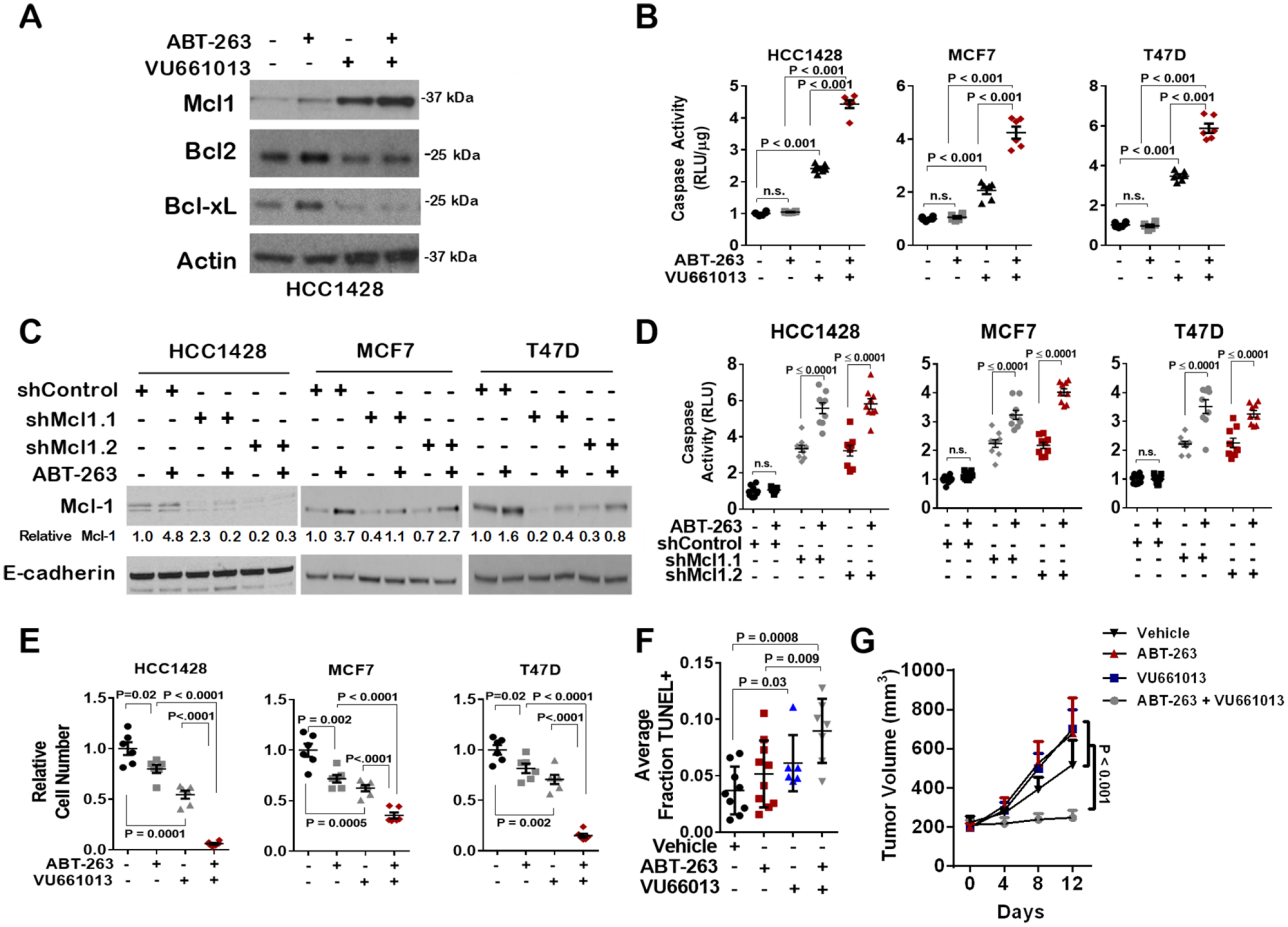
Increased Mcl-1 activity, but not expression, in response to ABT-263 is blocked by the novel Mcl-1 inhibitor VU661013 **(A)** Western analysis of whole cell lysates harvested from cells treated with 1 µM ABT-263 and/or 1 µM VU661013 for 16 hrs. Antibodies used for western analysis are shown at left. **(B)** Caspase 3/7 activity was measured in cells treated with 1 µM VU661013 and/or 1 µM ABT-263 for 16 hrs. Data points are the average RLUs corrected for total protein in three technical replicates, midlines are the average RLUs corrected for total protein of 3-6 biological replicates (±S.E.). The average RLUs in control cells for each cell line was set at a value of 1, *Two-way ANOVA*. **(C)** Western analysis of cells expressing shControl or shMcl-1, treated with ABT-263 (1 µM) or with DMSO. Antibodies used are shown to left of each panel. Relative Mcl-1 expression was determined using densitometry analysis. **(D)** Caspase 3/7 activity was measured in cells expressing shControl or shMcl1 shRNA sequences, and treated with 1 µM ABT-263 for 4 hrs. Data points are the average RLUs corrected for total protein in three technical replicates, midlines are the average RLUs corrected for total protein of 6-9 biological replicates (±S.E.). The average RLUs in control cells for each cell line was set at a value of 1, Two-way ANOVA. **(E)** Cells were grown for 7 days with 1 µM VU661013 and/or 1 µM ABT-263. Average relative number of cells per well is shown, N = 6-10, Two-way ANOVA followed by Tukey's multiple comparison's test. **(F–G)** MCF7 tumor xenografts in athymic mice were treated with ABT-263 (20 mg/kg, once daily) and/or VU661013 (25 mg/kg, once weekly) for 12 days. TUNEL analysis was used to detect apoptotic cells in tumors collected on treatment day 12, one hour after final treatment (F). Each data point represents the percentage of TUNEL+ cells in 3 random fields per sample, N = 6-10 per group. Tumor volume of MCF7 xenografts were measured once every four days beginning on treatment day 0 (G). Average tumor volume (S.E.) is shown. N = 6-10.

We measured Mcl-1 protein levels in cells treated with VU661013, finding increased Mcl-1 protein expression after 16 hr (Figure 5A). As expected, Mcl-1 expression was also upregulated upon treatment with ABT-263 and remained high in cells treated with the combination of ABT-263 and VU661013. Interestingly, Bcl-xL and Bcl-2 levels were not elevated in cells treated with ABT-263 + VU661013. While Caspase 3/7 activity was increased upon treatment with VU661013, we found that caspase 3/7 activity was further increased when cells were treated with the combination of ABT-263 + VU661013 (Figure 5B). These data are consistent with what was seen in ER+ breast cancer cells expressing shRNA sequences against Mcl-1 and treated with ABT-263 (Figure 5C), which increased caspase 3/7 activity to a greater extent than Mcl-1 knockdown alone or ABT-263 treatment alone (Figure 5D). Importantly, the combination of ABT-263 with VU661013 potently inhibited growth of tumor cells cultured in monolayer (Figure 5E) similar to the growth inhibition seen with ABT-263 in combination with shRNA sequences knocking down Mcl-1 (Supplementary Figure 6).

The growth inhibitory effects of VU661013 were confirmed *in vivo* using MCF7 xenografts grown in athymic mice. Tumor-bearing mice were treated daily with ABT-263 (20 mg/kg) alone, or in combination with once weekly treatment with VU661013 (25 mg/kg). Tumor cell death assessed by TUNEL analysis revealed a two-fold increase in tumor cell death in samples treated with VU661013 as a single agent (Figure 5F and Supplementary Figure 7). Although tumors treated with single agent ABT-263 did not display statistically increased tumor cell death, tumor cell death was markedly increased in tumors treated with the combination of ABT-263 and VU661013. Tumor volume measurements showed that, while ABT-263 treatment had little impact on tumor volume, VU661013 decreased tumor volume by approximately 25% versus control-treated tumors (Figure 5G). However, the combination of ABT-263 with VU661013 blocked tumor growth, supporting the hypothesis that Mcl-1 targeting improves treatment response of ER+ breast tumors to ABT-263, and highlighting the potential utility of Mcl-1 inhibitors in ER+ breast cancers.

## DISCUSSION

The anti-apoptotic Bcl-2 family member Mcl-1 is highly expressed in all breast cancer subtypes, although relatively little is known about Mcl-1 in breast tumor biology. With the ongoing development of Mcl-1 specific BH3-mimetics [28, 29], a better understanding of the role of Mcl-1 in breast cancer is imperative. A growing body of data suggests that Mcl-1 may play a strategic role in ERα+ breast cancers, a breast cancer subtype that expresses Bcl-2, Bcl-xL and Mcl-1. Importantly, Mcl-1 is a key survival factor used by several cancers to promote cell survival upon inhibition of Bcl-2 and Bcl-xL, including ER+ breast cancers [17, 30]. We report here that mTOR-dependent *MCL1* translation rapidly drives Mcl-1 upregulation in ER+ breast cancer cells treated with ABT-263, or with BH3 mimetics that target Bcl-2 alone (ABT-199) or Bcl-xL alone (A1155463), consistent with the well-established oncogenic role of mTOR in ER+ breast cancers [31, 32]. Also, these findings are supported by reports in other cancers suggesting that cancer cells often use rapid Mcl-1 translation to evade therapeutically-induced tumor cell death [33–35]. Previous studies in colon cancers [36], rhabdomyosarcomas [37], lung cancers [38, 39], glioblastomas [40], and ovarian cancers [41] demonstrated that under conditions of cellular duress, including treatment with ABT-263, Mcl-1 protein levels are induced downstream of mTOR signaling. A recent study examining *PIK3CA*-mutant breast cancers showed that heightened PI3K-mTOR signaling is a key driver of Mcl-1 overexpression in these tumor cells, and that PI3K/mTOR inhibition impaired Mcl-1 expression, increasing *PIK3CA*-mutant cell sensitivity to ABT-263 [17]. Studies presented herein support a wider impact of mTORC1 signaling on Mcl-1 expression, highlighting the mTORC1-to-Mcl-1 axis in *PIK3CA*-wild type ER+ breast cancer cells (e.g. HCC1428). Additionally, we found that mTOR inhibition, even in the absence of ABT-263, effectively reduced Mcl-1 expression, and induced apoptosis in ER+ breast cancer cells, suggesting that Mcl-1 diminution may contribute to the molecular mechanisms of cell death and growth inhibition in ER+ breast cancers treated with RAD001. While this idea requires further exploration, this hypothesis is interesting to note in light of the recently published case study in which a patient with late stage *MCL1*-amplified ERα+ breast cancer treated with aromatase inhibition (anastrozole) and RAD001/everolimus achieved a pathological complete response [42].

While the results shown here suggest that Mcl-1 may be targeted indirectly using mTORC1 inhibitors, there is much excitement over the emerging class of selective Mcl-1 inhibitors. Here, we assessed the Mcl-1 inhibitor VU661013 in ER+ breast cancer cells, finding that VU661013 induced caspase activity, inhibited growth, and induced apoptosis of ER+ breast cancer cells in culture and *in vivo*. Further, we report that the combined inhibition of Mcl-1 using VU661013 with blockade of Bcl-2/Bcl-xL using ABT-263 resulted in superior tumor cell killing and tumor growth inhibition in ERα+ breast cancers in culture and *in vivo*. We confirmed that VU661013 blocked Mcl-1 interactions with Bim *in situ* in ER+ breast cancer cells using PLA. Interestingly, VU661013-treated ER+ breast cancer cells upregulated Mcl-1 protein levels, as shown by western analysis, although PLA confirmed that Mcl-1 interactions with Bim remained suppressed by VU661013. Mcl-1 upregulation in response to the Mcl-1 inhibitor S68345 has been reported previously in *HER2*-amplified breast cancers and TNBC [2, 43]. In contrast to Mcl-1 upregulation upon treatment with VU661013, we did not see upregulation of Bcl-2 or Bcl-xL in ER+ breast cancer cells treated with the Mcl-1 inhibitor. This may suggest that, in ER+ breast cancers, Mcl-1 plays a dominant role among Bcl-2 family members under conditions in which an imbalance of pro- and anti-apoptotic factors threaten cell survival. This hypothesis would require further investigation, but is supported by observations that the *MCL1* gene is more frequently amplified and overexpressed in ER+ breast cancers as compared to genes encoding all other Bcl-2 family members combined [11].

In summary, we report here that mTOR-dependent translation of Mcl-1 increases Mcl-1 expression and promotes cell survival in ER+ breast cancer cells. This signaling is elevated in cells treated with the Bcl-2/Bcl-xL inhibitor ABT-263, but is diminished upon inhibition of mTORC1 using RAD001. Importantly, we have tested the Mcl-1 selective inhibitor VU661013 in ER+ breast cancer cells, finding that Mcl-1 inhibition increases apoptosis and decreases tumor cell death, particularly when used in combination with ABT-263. These studies support further investigation of VU661013 and other emerging Mcl-1 inhibitors in ER+ breast cancers.

## MATERIALS AND METHODS

### Cell culture

All cell lines used in these studies were purchased directly from American Tissue Type Collection (*Homo sapiens* ATCC CRL 2327; HTB-22; HTB-133), and cultured in growth media [DMEM (Corning Cellgro), 10% fetal bovine serum (**FBS**, Life Technologies), 1x antibiotics/anti-mycotics (Life Technologies)]. Mcl-1 expression was stably ablated used lentiviral particles containing two separate shRNA sequences against Mcl-1 (shMCL1), and a scramble control (shControl), according to the manufactures protocol (Santa Cruz Biotechnology, SC-35878-V) and as described previously. Cells were selected with puromycin (Life Technologies). Growth was measured by seeding 2,500 cells in a 35-mm dish, treating with inhibitors (1.0 µM ABT-263, 200 nM RAD001, 1 µM VU661013) in growth media after the cells adhered for 16 hr. Caspase-3/7 activity was measured using Caspase-3/7 Glo (Promega). 10,000 cells/well plated in a 96-well dish were treated the following day ± 1.0 µM ABT-263, 200 nM RAD001, or 1 µM VU661013 for 4 hr or 48hr. Mcl-1 protein stability was determined after treatment with 1.0 µM ABT-263 for 16 hr and treatment with CHX (50 µg/mL, Sigma-Aldrich) for the final 0, 15, and 30 min. Whole cell lysates were harvested and analyzed by western analysis according to the above protocol.

### RT-qPCR analysis

RT-qPCR was completed as previously described [44], using human *MCL1* primers [Integrated DNA Technologies, Forward (5’-CCTTCCAAGGATGGGTTTGTGGA) and Reverse (5’-TGCCACTTGCTT TTCTGGCT)], Human *36B4* control primers (Integrated DNA Technologies). ∆∆C_t_ values were standardized to *36B4* and the average standardized ∆∆C_t_ values were presented.

### Western analysis and immunoprecipitation

For western analysis, whole cell lysates were harvested in ice-cold NP-40 lysis buffer (**NLB**) [50 mM Tris pH 7.4, 100 mM NaF, 120 mM NaCl, 0.5% NP-40, 100 µM Na_3_VO_4_, 1X protease inhibitor cocktail (Roche), 0.5 µM proteasome inhibitor (Santa Cruz Technologies)]. Protein concentration was determined by BCA assay (Pierce) and 20 µg protein per sample were resolved on 4-12% sodium dodecyl sulfate (SDS)-polyacrylamide gel electrophoresis (PAGE) gels (NuPAGE, InVitrogen). For immunoprecipitation, protein A/G agarose beads (Santa Cruz Biotechnology) were cross-linked with 10 μg antibody (as indicated) using 5 mM BS3 (Sigma-Aldrich) at room temperature for 30 min, quenched using 15 µL 1.0 M Tris (pH 7.4), washed with ice-cold NLB, diluted 1:4 in phosphate buffered saline (PBS, pH 7.4). 10µl conjugated antibody-beads incubated overnight with 1000 µg whole cell lysates collected in NLB. Beads were washed six times with 0.25X NLB, boiled in 1x reducing sample buffer (NuPAGE, Invitrogen), and resolved on a 4-12% SDS-PAGE gel as detailed in [11]. Proteins were transferred to nitrocellulose (iBlot, Invitrogen), blocked in 3% gelatin (Sigma-Aldrich) in Tris buffered saline (**TBS**), 0.1% Tween-20 (Sigma-Aldrich), then probed with primary antibodies [Bim (1:500), Bcl-xL (1:500), and β-actin (1:10,000) from Cell Signaling Technology); Mcl-1 (1:500) from Santa Cruz Biotechnology; and Bcl-2 (1:1000) from DAKO], and secondary antibodies [horseradish peroxidase-conjugated donkey anti-rabbit (1:10,000) or donkey anti-mouse (1:5000), Sigma-Aldrich] and developed using enhanced chemi-luminescence (Pierce).

### Murine models

Mice were housed under pathogen-free conditions and all experiments were in accordance with AAALAC guidelines and with Vanderbilt University Institutional Animal Care and Use Committee approval. MCF7 xenografts were generated as described previously [45]. Tumor volume was measured every other day once tumors became palpable. Treatment of mice began when tumors reached 200 mm^3^. Mice were treated with once daily with oral RAD001 (2 mg/kg), oral ABT-263 (50 mg/kg), or treated once weekly with VU661013 (25 mg/kg), by intraperitoneal injection in 50 µl vehicle (2.5% DMSO, 50% polyethylene glycol (PEG)-300, 47.5% sterile saline).

### Histological analysis

All samples were fixed in 10% formalin, paraffin-embedded and 5-μm sections were stained with TUNEL using the ApopTag kit (Calbiochem). IHCfor Mcl-1, phospho-p70-S6K (Cell Signaling Technologies) and phospho-histone H3 (Santa Cruz Biotechnology) were performed on sections as described [45, 46]. All histological analyses were photographed using Olympus DP2 software (200x).

### Proximity ligation assay (PLA)

Cells cultured in 96-well plates were fixed with methanol and Duolink (Sigma) proximity ligation protocol was used according to manufacturer’s directions using Mcl-1 (Santa Cruz Biotechnology, 1:25) and Bim (Santa Cruz Biotechnology, 1:25) antibodies. Cells were counterstained with Hoescht. Plates were scanned by ImageXpress Micro XL Automated Microscope. PLA fluorescent puncta were enumerated using the automated Duolink ImageTool. Puncta per cell values were recorded for >20 cells per 400x field.

### Statistical analysis

Statistical significance (P<0.05) was determined using Student’s unpaired two-tailed *T*-test or ANOVA with Tukey’s Multiple Comparisons test, using the Graphpad Prism 6 software. For animal studies significance was determined by area under the curve.

## SUPPLEMENTARY MATERIALS FIGURES



## Abbreviations

ER: estrogen receptor
TNBC: triple negative breast cancer
BH3: Bcl-1 homology-3
Hr: hours
qRT-PCR: quantitative real time polymerase chain reaction
CHX: cycloheximide
PI3K: phosphatidyl inositol-3 kinase
mTORC1: mTOR Complex 1
4EBP1: 4-Elongating Initiating Factor Binding Protein-1
PLA: Proximity Ligation Assay
IHC: Immunohistochemistry
S6K: S6-kinase
TUNEL: Teminal dUTP Nick End Labeling
shRNA: short hairpin RNA
FBS: Fetal Bovine Serum
NLB: Nonident P-40 Lysis Buffer
SDS PAGE: Sodium Dodecyl Sulfate Polyacrylamide Gel Electrophoresis
IP: Immuno-precipitation
PBS: Phosphate Buffered Saline
TBS: Tris buffered Saline.
