# Supplementary material for: Therapeutic inhibition of Mcl-1 blocks cell survival in estrogen receptor-positive breast cancers
Source: Oncotarget. 2019 Oct 9;10(52):5389–402. doi: 10.18632/oncotarget.27070 (PMC6739218; doi:10.18632/oncotarget.27070)
Supplement: Supplementary file 1 [file oncotarget-10-5389-s001.pdf]

Therapeutic inhibition of Mcl-1 blocks cell survival in estrogen receptor-positive breast cancers

SUPPMENTARY MATERIALS

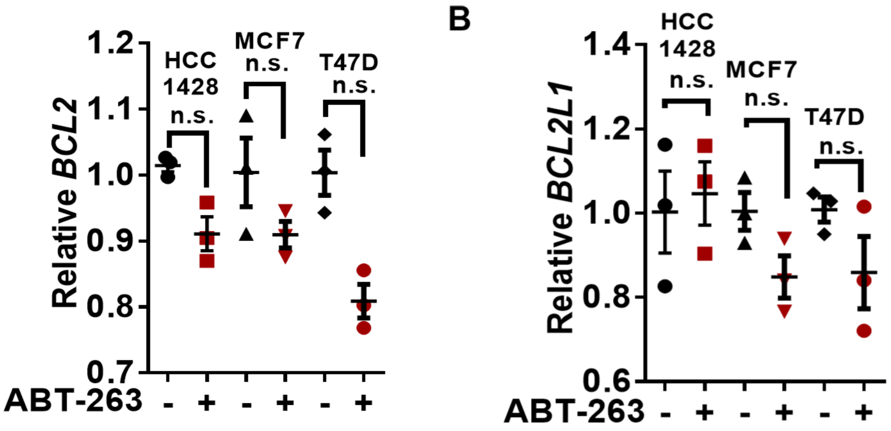

Supplementary Figure 1: *BCL2* and *BCL2L1* transcript levels are unaffected by ABT-263 treatment in ER+ breast cancer cells. RT-PCR was used to measure *BCL2* **(A)** or *BCL2L1* **(B)** relative transcripts in cells treated for 6 hours with ABT-263 (1 μM) in full growth medium.

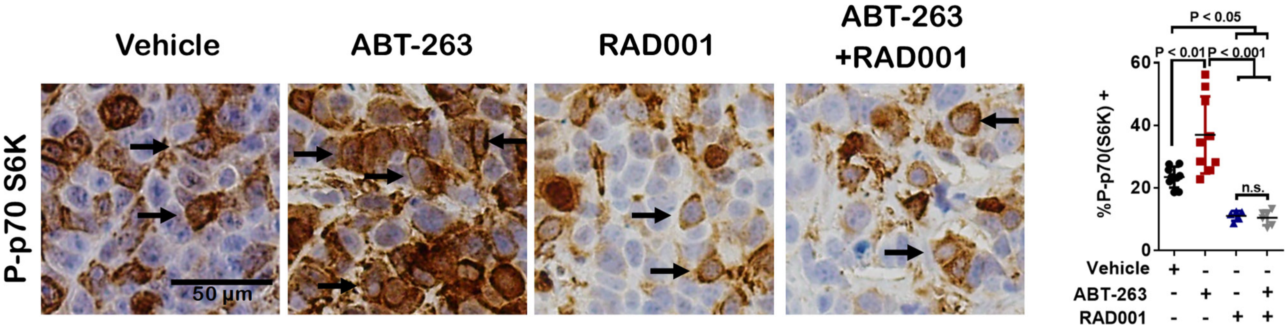

Supplementary Figure 2: Increased mTORC1 activity in tumors treated with ABT-263. Phospho-p70 S6K (P-p70 S6K) IHC staining completed in MCF7 xenografts after treatment with 20 mg/kg ABT-263 ± 1.0 mg/kg RAD001 daily by oral gavage for 16 d. Left + representative images at 400X (arrows denote positive cells). Right = quantitation of P-P70 S6K staining. Values represent average number of P-p70S6K positive cells per total nuclei in three fields of view per tumor. N = 6-10 tumors. Statistical significance tested by ANOVA followed by Tukey's multiple comparison's test.

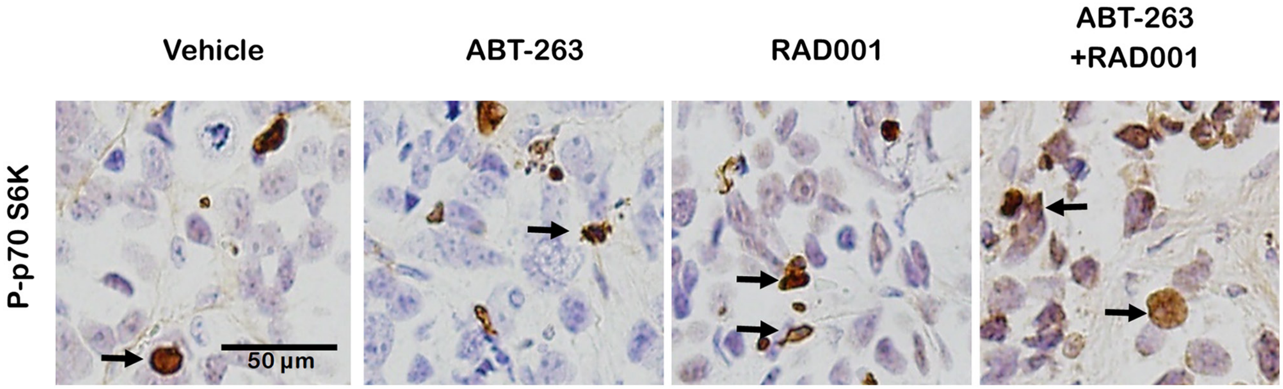

Supplementary Figure 3: mTORC1 inhibition increases cell death in ER+ breast tumors treated with ABT-263. TUNEL staining of MCF7 xenografts grown in mice treated with 20 mg/kg ABT-263 ± 1.0 mg/kg RAD001 by oral gavage for 21 days. Representative images collected at 400X magnification are shown. Arrows denote positive cells.

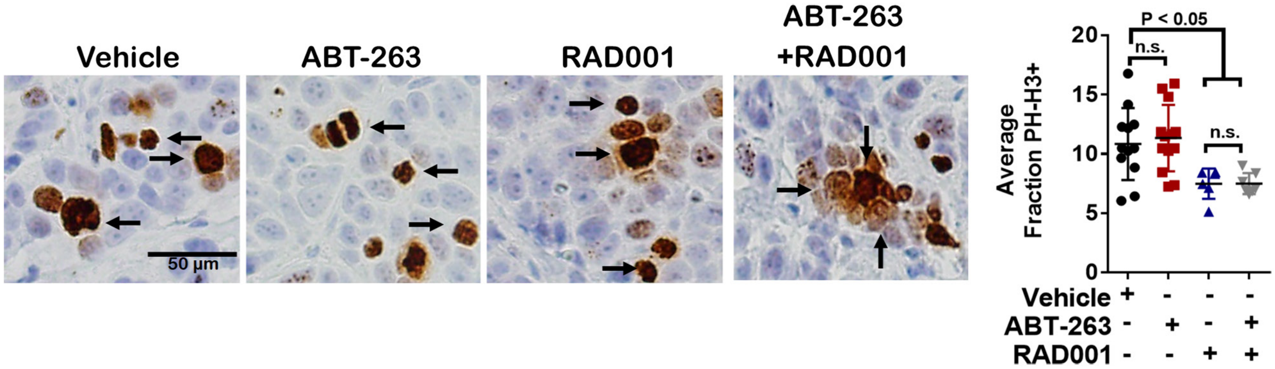

Supplementary Figure 4: Cell proliferation in unaffected by ABT-263. Phospho-histone H3 (PH-H3) IHC staining was assessed in MCF7 xenografts treated with 20 mg/kg ABT-263 263 ± 1.0 mg/kg RAD001 by oral gavage for 21 days. Left panels shown representative 400X images, arrows denote positive cells. Right panels show quantitation of the average number of PH-H3+ cells per total nuclei in three random fields of view per tumor, N = 6-10 tumors per group. Statistical significance assessed with ANOVA followed by Tukey's multiple comparisons test.

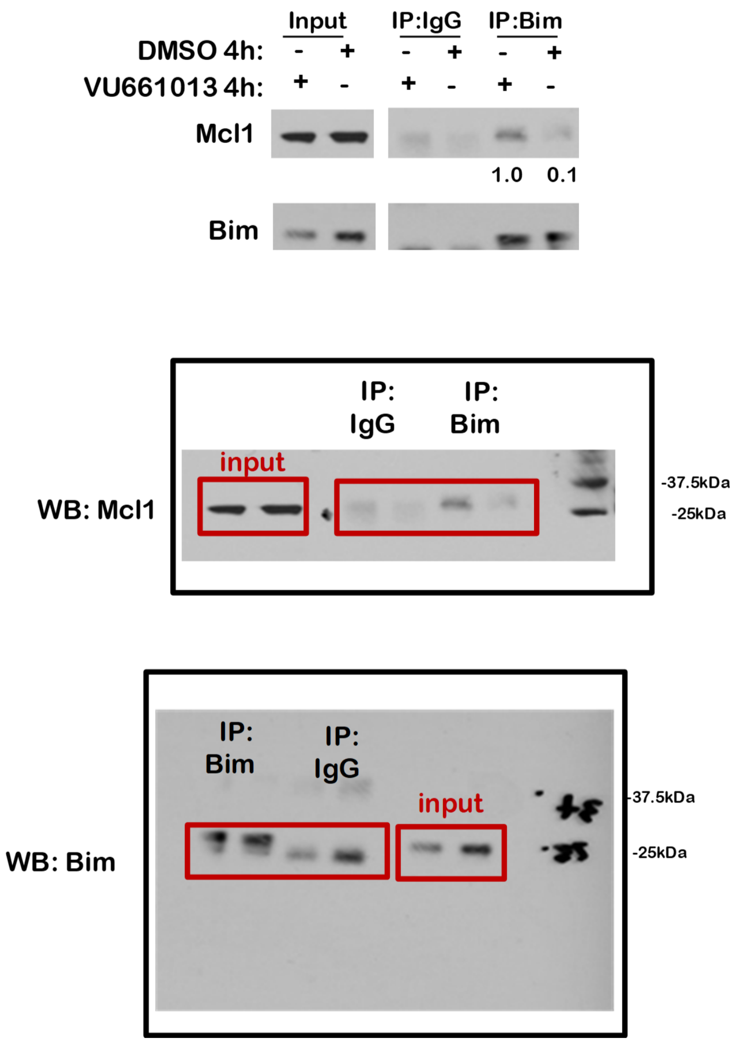

Supplementary Figure 5: Mcl-1 co-precipitation with Bim is diminished upon treatment of HCC1428 cells with Mcl-1 inhibitor VU661013. Input bands are indicated.

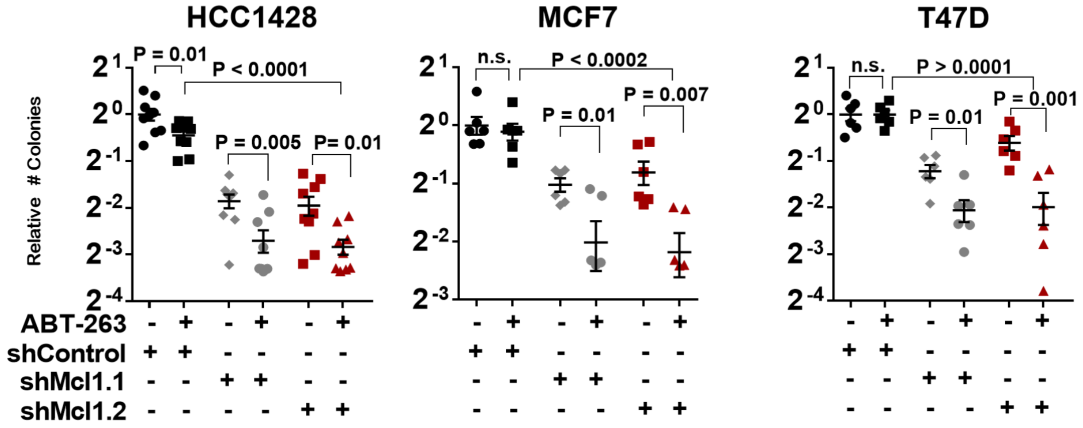

Supplementary Figure 6: Mcl-1 knockdown sensitizes ER+ breast cancer cells to ABT-263. Equal number of cells were plated on day 1 in full growth media. Cells were cultured for 7 days. Cells were counted by trypan blue exclusion using the TC-10 automated cell counter. Average value for DMSO-treated cells expressing shControl sequences was set at a value of 1 for each cell line. All other values are expressed relative to the average value for controls. Each data point represents the average of two experimental replicates. Midlines represent the average of all biological replicates (± S.E.). Statistical significance assessed with ANOVA followed by Tukeys multiple comparisons test.

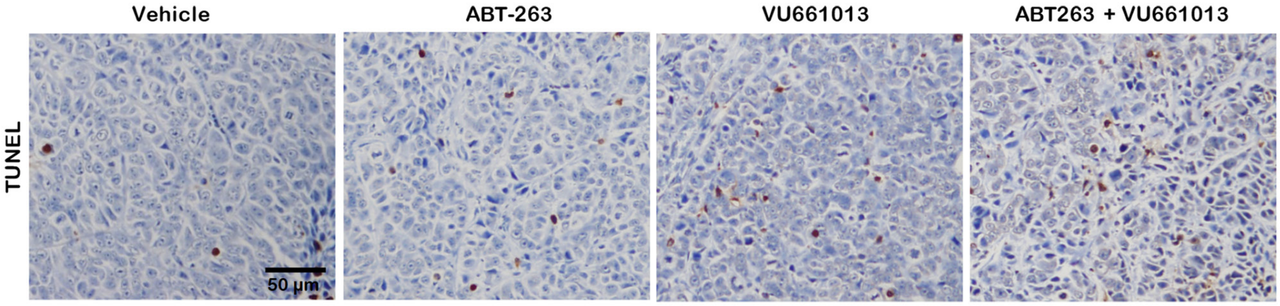

Supplementary Figure 7: VU661013 increases breast cancer cell death *in vivo*. TUNEL-stained MCF7 xenografts after treatment with 20 mg/kg ABT-263 (daily) ±25 mg/kg VU661013 (once weekly) for 12 days.
